# The interplay between age at menopause and synaptic integrity on Alzheimer’s disease risk in women

**DOI:** 10.1126/sciadv.adt0757

**Published:** 2025-03-05

**Authors:** Madeline Wood Alexander, William G. Honer, Rowan Saloner, Liisa A. M. Galea, David A. Bennett, Jennifer S. Rabin, Kaitlin B. Casaletto

**Affiliations:** ^1^ Hurvitz Brain Sciences Program, Sunnybrook Research Institute, Toronto, ON, Canada.; ^2^ Rehabilitation Sciences Institute, Temerty Faculty of Medicine, University of Toronto, Toronto, ON, Canada.; ^3^ Department of Psychiatry, University of British Columbia, Vancouver, BC, Canada.; ^4^ Memory and Aging Center, Department of Neurology, Weill Institute for Neurosciences, University of California, San Francisco, CA, USA.; ^5^ Department of Pharmacology & Toxicology, University of Toronto, Toronto, Ontario, Canada.; ^6^ Campbell Family Mental Health Research Institute, The Centre for Addition and Mental Health, Toronto, Ontario, Canada.; ^7^ Department of Psychiatry, Temerty Faculty of Medicine, University of Toronto, Toronto, Ontario, Canada.; ^8^ Rush Alzheimer's Disease Center, Rush University Medical Center, Chicago, IL, USA.; ^9^ Centre for Brain Resilience and Recovery, Hurvitz Brain Sciences Program, Sunnybrook Research Institute, Toronto, ON, Canada.; ^10^ Division of Neurology, Temerty Faculty of Medicine, Sunnybrook Health Sciences Centre, University of Toronto, Toronto, ON, Canada.; ^11^ Harquail Centre for Neuromodulation, Sunnybrook Health Sciences Centre, University of Toronto, Toronto, ON, Canada.

## Abstract

Menopause is a major biological transition that may influence women’s late-life brain health. Earlier estrogen depletion—via earlier menopause—has been associated with increased risk for Alzheimer’s disease (AD). Synaptic dysfunction also incites and exacerbates AD progression. We investigated whether age at menopause and synaptic health together influence AD neuropathology and cognitive trajectories using clinical and autopsy data from 268 female decedents in the Rush Memory and Aging Project. We observed significant interactions between age at menopause and synaptic integrity on cognitive decline and tau tangles, such that earlier menopause strengthened the associations of reduced synaptic integrity with faster cognitive decline and elevated tau. Exploratory analyses showed that these relationships were attenuated in women who took menopausal hormone therapy. These findings suggest that midlife endocrine processes or their sequalae may influence synaptic vulnerability to AD. Interventions addressing both hormonal factors and synaptic health could enhance resilience to dementia in women.

## Teaser

Earlier age at menopause exacerbates synapse-related vulnerability to cognitive decline and tau tangles.

## INTRODUCTION

Two-thirds of individuals diagnosed with Alzheimer’s disease (AD) dementia are women, but the causes of this sex disparity remain largely unknown (*1*–*3*). Accumulating data suggest that biological sex plays a fundamental role in the neuropathological and clinical manifestations of AD. Relative to men, women show greater burdens of tau at equivalent levels of β amyloid (*4*, *5*) and experience faster cognitive decline at comparable levels of AD pathology (i.e., β amyloid and tau), particularly after symptom onset (*6*, *7*).

Menopause may contribute to women’s increased risk of AD. This natural biological process involves a substantial decline in ovarian hormones, including estradiol, which has neuroprotective effects. It also involves rises in gonadotropin hormones [e.g., follicle stimulating hormone (FSH)], which may have neurotoxic effects (*8*–*10*). Animal models of the menopause transition show that declines in estradiol trigger deleterious changes in synaptic functioning that could increase susceptibility to AD (*11*, *12*). Further, both loss of estradiol and increased FSH have been shown to promote AD pathology (*9*, *13*, *14*). Notably, earlier age at menopause, which results in shorter lifetime exposure to estradiol, has been linked to greater tau burden and an increased risk for AD dementia (*15*–*18*). Exposure to exogenous estrogens via menopausal hormone therapy may attenuate the impacts of earlier menopause on AD cognitive and brain outcomes, but findings have been inconsistent (*19*).

Synaptic loss is an early and prominent neuropathological feature of AD that occurs before fulminant plaques and tangles and strongly correlates with cognitive decline (*20*–*23*). Thus, preservation of synaptic integrity may protect against AD dementia. Consistent with this hypothesis, studies show that among individuals with AD pathology, those with intact cognitive function have better synaptic health compared to those with cognitive impairment (*24*, *25*). Furthermore, in vivo and autopsy studies suggest that synaptic integrity influences relationships within the amyloid-tau-neurodegeneration cascade. Specifically, mixed-sex research reports that older adults with greater synaptic dysfunction show disproportionate tau accumulation relative to their level of β amyloid and disproportionate brain atrophy relative to their tau burdens (*26*). Together, these data suggest that synaptic integrity may provide resistance against AD propagation and resilience to cognitive decline once pathology is present (*26*, *27*).

Despite the known role of estrogens in synaptogenesis (*28*), there remains a notable lack of research investigating how women’s endocrine health factors influence the interplay between β amyloid, tau, and synaptic functioning. This study aimed to address this gap by examining how menopause-related endocrine factors—such as age at menopause and hormone therapy—associate and interact with biomarkers of synaptic integrity to influence cognitive decline and post-mortem AD neuropathology. To do so, we leveraged clinical and autopsy data from female decedents in the Rush Memory and Aging Project (MAP). This included brain tissue levels of synaptic proteins abundant on inhibitory (complexin-I) and excitatory (complexin-II) presynaptic terminals, as well as a composite measure reflecting levels of protein-protein interactions at the soluble *N*-ethylmaleimide–sensitive factor attachment protein receptor (SNARE) complex [i.e., synaptosomal-associated protein 25 (SNAP-25), vesicle-associated membrane protein (VAMP), and syntaxin-1], which has critical roles in neurotransmitter release. We tested the hypotheses that earlier age at menopause would strengthen the associations of reduced synaptic integrity with post-mortem measures of AD pathology (i.e., β amyloid and tau tangles) and longitudinal antemortem cognitive decline. In addtion, as an exploratory objective, we investigated whether these effects were attenuated in women with a history of menopausal hormone therapy.

## RESULTS

### Participant characteristics

The main analyses included 268 women who reported experiencing spontaneous menopause. Exploratory analyses examining the influence of menopausal hormone therapy on outcomes of interest included 264 participants with available data for lifetime hormone therapy use (history of menopausal hormone therapy use versus non-use). Demographic and clinical characteristics are summarized in Table 1. The mean (SD) age at menopause in the main analytic sample was 49.2 (4.89) years. Figures S1 and S2 depict frequency histograms and density plots of age at menopause in the main analytic sample and in the exploratory sample for hormone therapy analyses, respectively.

**Table 1.** Participant characteristics. HT, hormone therapy; MCI, mild cognitive impairment; AD, Alzheimer’s disease. *P* values reflect the results of Welch two-sample *t* tests (for age at baseline, complexin-I, and complexin-II), Wilcoxon rank sum tests (for remaining continuous variables), and χ^2^ tests (for categorical variables) comparing women with versus without history of HT. *P < 0.05, **P < 0.01, and ***P < 0.001.

	Main analytic sample	Exploratory HT sample	
	All participants, *N* = 268	Women with history of HT, *N* = 75	Women with no HT, *N* = 189
Age at baseline, mean (SD) years	83.7 (5.88)	82.0 (5.68)**	84.4 (5.83)**
Age at death, mean (SD) years	90.9 (5.90)	89.2 (5.65)***	91.6 (5.91)***
Years of education, mean (SD)	14.2 (2.55)	14.1 (2.52)	14.2 (2.56)
*APOE* ε4 carriage, *N* (%)	58 (21.6)	13 (17.3)	43 (22.8)
Race/ethnicity			
White, N (%)	264 (98.5)	74 (98.7)	186 (98.4)
Black or African American, N (%)	3 (1.11)	0 (0)	3 (1.59)
American Indian or Alaska Native, N (%)	1 (0.37)	1 (1.33)	0 (0)
Age at menopause, mean (SD) years	49.2 (4.89)	48.9 (5.22)	49.4 (4.77)
Number of annual clinical visits, median (SD)	7 (3.88)	7 (4.09)	7 (3.79)
Final clinical diagnosis			
No cognitive impairment, N (%)	88 (33.8)	34 (45.3)*	53 (28.0)*
MCI, N (%)	72 (26.9)	18 (24.0)	53 (28.0)
AD dementia, N (%)	88 (32.8)	18 (24.0)	69 (36.5)
Other (non-AD) dementia, N (%)	4 (1.49)	2 (2.67)	2 (1.06)
Post-mortem neuropathological markers			
Complexin-I^†^, mean (SD)	0.08 (0.83)	0.13 (0.86)	0.05 (0.82)
Complexin-II^†^, mean (SD)	0.09 (0.78)	0.14 (0.77)	0.08 (0.78)
SNARE protein-protein interactions†, mean (SD)	0.03 (0.83)	0.09 (0.79)	−0.003 (0.83)
SNARE density^†^, mean (SD)	0.04 (0.89)	0.06 (0.89)	0.03 (0.90)
β amyloid^‡^, mean (SD)	4.62 (4.36)	4.13 (4.15)	4.81 (4.42)
Tau tangles^§^, mean (SD)	7.43 (8.08)	6.11 (6.90)*	7.79 (8.21)*
Age started HT, mean (SD) years	–	51.7 (12.5)	–
Age stopped HT, mean (SD) years	–	63.4 (13.9)	–
HT duration, mean (SD) years	–	11.8 (10.9)	–
HT initiation relative to menopause	–		–
Within 5 years post-menopause, N (%)	–	41 (54.7)	–
After 5 years post-menopause, N (%)	–	9 (12.0)	–
HT type	–		–
Pill, N (%)	–	65 (86.7)	–
Patch, N (%)	–	4 (5.33)	–
Cream or suppository, N (%)	–	11 (14.7)	–
Injection, N (%)	–	6 (8.00)	–

†Synaptic biomarkers are expressed in log_10_ units, standardized, and averaged across six brain regions.

‡β amyloid is expressed in percent area of the cortex occupied, averaged across eight brain regions.

§Tau tangles are expressed in cortical density (per square millimeter), averaged across eight brain regions.

### Independent associations between age at menopause, synaptic biomarkers, and AD pathology

Before addressing our primary research questions, we performed a series of analyses to examine independent associations between our key variables. Age at menopause was not significantly associated with brain tissue biomarkers of synaptic integrity or AD neuropathology [complexin-I: β = −0.007, 95% confidence interval (CI) = −0.028 to 0.013, *P* = 0.47; complexin-II: β = −0.003, 95% CI = −0.023 to 0.016, *P* = 0.72; SNARE protein-protein interactions: β = −0.001, 95% CI = −0.021 to 0.019, *P* = 0.90; β amyloid: β = −0.012, 95% CI = −0.039 to 0.014, *P* = 0.36; and tau tangles: β = 0.006, 95% CI = −0.022 to 0.034, *P* = 0.68]. These findings suggest that age at menopause does not relate to postmortem markers of synaptic integrity or AD neuropathology decades following menopause.

Levels of complexin-II (β = −0.202, 95% CI = −0.366 to −0.039, *P* = 0.02) and SNARE protein-protein interactions (β = −0.160, 95% CI = −0.319 to −0.0004, *P* = 0.049) were inversely associated with β amyloid. The association of complexin-I with β amyloid was not significant (β = −0.123, 95% CI = −0.280 to 0.033, *P* = 0.12). Levels of complexin-I (β = −0.102, 95% CI = −0.271 to 0.067, *P* = 0.24), complexin-II (β = 0.075, 95% CI = −0.104 to 0.255, *P* = 0.41), and SNARE protein-protein interactions (β = −0.132, 95% CI = −0.304 to 0.041, *P* = 0.13) were not significantly associated with tau tangles.

### Synergistic associations between age at menopause and synaptic biomarkers on AD neuropathology

Age at menopause did not moderate the association between any of the synaptic biomarkers levels and β amyloid (complexin-I: β = 0.023, 95% CI = −0.012, 0.057; *P* = 0.21; complexin-II: β = 0.018, 95% CI = −0.022, 0.058; *P* = 0.37; and SNARE protein-protein interactions: β = 0.025, 95% CI = −0.009, 0.059; *P* = 0.16).

**Fig. 1.**
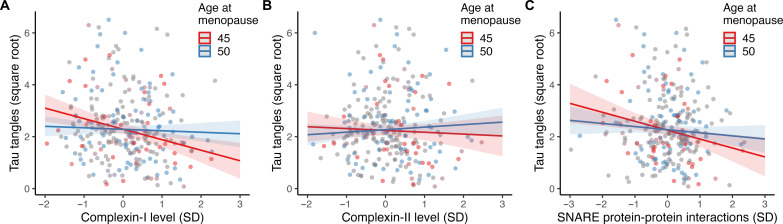
Synergistic associations of age at menopause and synaptic biomarkers with tau tangles. The plots depict separate models of the two-way interactions between age at menopause and levels of (**A**) complexin-1, (**B**) complexin-II, and (**C**) SNARE protein-protein interactions on tau tangles. The models are adjusted for age at death, years of education, β amyloid, *APOE* ε4 carriage, and mean SNARE density [for (C) only]. Age at menopause was modelled continuously, and the model estimates for earlier (i.e., age 45) and average (i.e., age 50) ages at menopause are shown for visualization purposes. Shaded regions represent 95% CIs.

However, age at menopause significantly moderated the association between synaptic biomarker levels and tau tangles, adjusting for β amyloid levels. Specifically, earlier age at menopause exacerbated the negative associations of synaptic dysfunction on tau. As depicted in Fig. 1, this moderation effect was observed for complexin-I (β = 0.070, 95% CI = 0.033, 0.106; *P* = 0.0002) and SNARE protein-protein interactions (β = 0.045, 95% CI = 0.008, 0.081; *P* = 0.02). By contrast, no significant moderation effect was found for complexin-II (β = 0.034, 95% CI = −0.010, 0.077; *P* = 0.13).

### Associations between age at menopause, synaptic biomarkers, and global cognitive decline

We first estimated the main effects of each synaptic biomarker on global cognitive decline. There were no significant associations of any synaptic biomarker on change in cognition over time (complexin-I: β = −0.003, 95% CI = −0.026, 0.019; *P* = 0.77; complexin-II: β = −0.002, 95% CI = −0.027, 0.023; *P* = 0.87; and SNARE protein-protein interactions: β = 0.006, 95% CI = −0.016, 0.027, *P* = 0.61). There was also no significant association of age at menopause with cognitive decline (β = −0.002, 95% CI = −0.006, 0.002; *P* = 0.28).

**Fig. 2.**
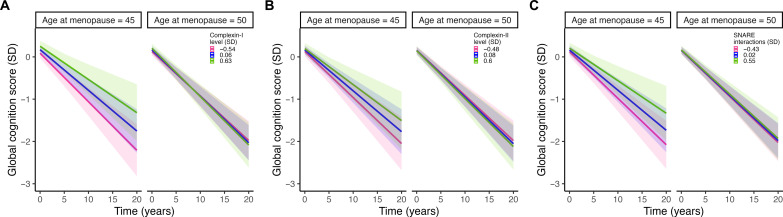
Synergistic associations of age at menopause and synaptic biomarkers on global cognitive decline. The plots depict separate models of the three-way interactions between age at menopause, levels of (**A**) complexin-1, (**B**) complexin-II, and (**C**) SNARE protein-protein interactions, and time on global cognitive scores. The models are adjusted for the interaction between age at death and time, along with years of education, *APOE* ε4 carriage, and mean SNARE density [for (C) only], and their interactions with time. Age at menopause and synaptic marker levels were both modelled continuously. For visualization purposes, model estimates are shown for earlier (i.e., age 45) and average (i.e., age 50) ages at menopause and for the 25th, mean, and 75th percentiles of each synaptic marker. Shaded regions represent 95% CIs.

After adjusting for relevant covariates, linear mixed models revealed significant three-way interactions between age at menopause, synaptic biomarkers, and time on cognitive decline. Specifically, at earlier ages of menopause, greater levels of synaptic dysfunction were associated with disproportionately steeper cognitive decline (Fig. 2). This pattern was observed for all the synaptic biomarkers examined (complexin-I: β = −0.008, 95% CI = −0.013, −0.003; *P* = 0.002; complexin-II: β = −0.006, 95% CI = −0.011, −0.0001; *P* = 0.047; and SNARE protein-protein interactions: β = −0.006, 95% CI = −0.010, −0.001; *P* = .01).

### Mediating effects of tau tangles: Post hoc analyses

In the above analyses, we demonstrated that women who experienced earlier menopause showed stronger associations between lower levels of synaptic biomarkers (i.e., complexin-I and SNARE protein-protein interactions) and both greater tau and faster cognitive decline. Building on these findings, we next investigated whether tau tangles may be a pathway by which synaptic integrity influences cognition in women with earlier menopause. To do so, we performed moderated mediation analyses to test the mediating effect of tau on the associations between each synaptic biomarker (i.e., complexin-I and SNARE protein-protein interactions) and cognitive decline at two ages of menopause: age 45 (i.e., earlier) and age 50 (i.e., average).

**Fig. 3.**
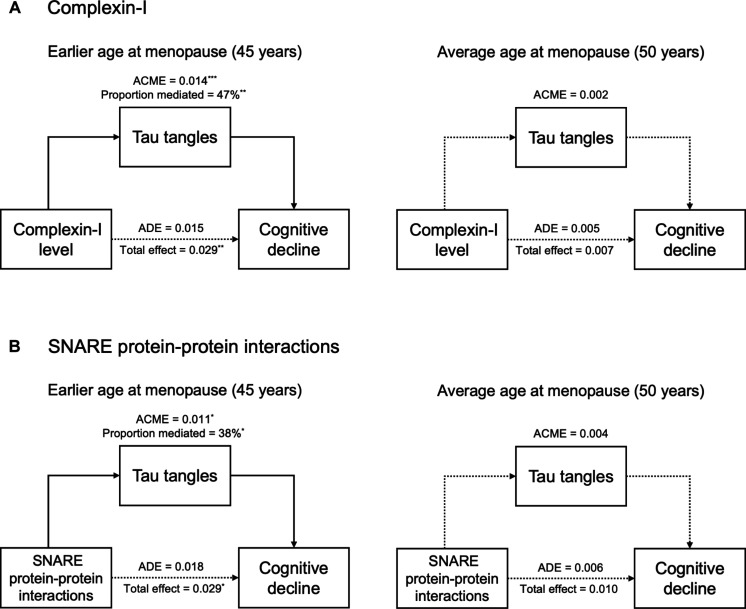
Tau tangles mediate the associations between synaptic biomarkers and global cognitive decline at earlier ages at menopause. Mediation analyses tested whether tau tangles mediate the associations of synaptic biomarkers with global cognitive decline at earlier and average ages at menopause. At earlier age at menopause (left), we observed significant tau-mediated effects of (**A**) complexin-I and (**B**) SNARE protein-protein interactions on cognitive decline. At average age at menopause (right), there were no significant tau-mediated effects of (A) complexin-I or (B) SNARE protein-protein interactions on cognitive decline. The models testing the interactions between synaptic markers and age at menopause on tau tangles are adjusted for age at death, years of education, β amyloid, *APOE* ε4 carriage, and mean SNARE density [for (B) only]. The models testing the effects of (i) the interactions between synaptic markers and age at menopause and (ii) tau tangles on cognitive slope are adjusted for the interaction between age at baseline and number of visits, years of education, β amyloid, *APOE* ε4 carriage, and mean SNARE density [for (B) only]. ACME, average causal mediation effect; ADE: average direct effect. *P < 0.05, **P < 0.01, and ***P < 0.001.

For complexin-I (Fig. 3A), tau tangles significantly mediated the association between complexin-I levels and cognitive decline at earlier age at menopause [average causal mediation effect (ACME) = 0.014, *P* < 0.0001] but not average age at menopause (ACME = 0.002, *P* = 0.51). Approximately 47% of the relationship between complexin-I and cognitive decline was explained by the level of tau tangles in women who experienced earlier age at menopause (*P* = 0.006). The direct association between complexin-I and cognitive decline was not significant in either earlier [average direct effect (ADE) = 0.015, *P* = 0.24] or average menopause (ADE = 0.005, *P* = 0.61).

A similar pattern was observed for SNARE protein-protein interactions (Fig. 3B). Specifically, tau tangles significantly mediated the association between SNARE protein-protein interaction levels and cognitive decline at earlier (ACME = 0.011, *P* = 0.02) but not average age at menopause (ACME = 0.004, *P* = 0.16). A total of 38% of the association between SNARE protein-protein interactions and cognitive decline was mediated by the level of tau tangles in women with earlier menopause (*P* = 0.03). The direct association between SNARE protein-protein interaction levels and cognitive decline was not significant in either earlier (ADE = 0.018, *P* = 0.08) or average menopause (ADE = 0.006, *P* = 0.46).

### Effects of hormone therapy: Exploratory analyses

Given the potential biological importance of menopausal hormone therapy, we performed exploratory analyses to investigate (i) if the independent effects of age at menopause on synaptic, tau, and cognitive outcomes differed by history of hormone therapy and (ii) if the observed synergistic interactions between age at menopause and synaptic biomarkers on tau and cognitive outcomes differed by history of hormone therapy.

With respect to independent associations, age at menopause was not significantly associated with synaptic biomarkers, AD neuropathology, or cognitive decline, either in women without history of hormone therapy or in women with history of hormone therapy (table S1). These findings align with those of the main analytic sample.

**Fig. 4.**
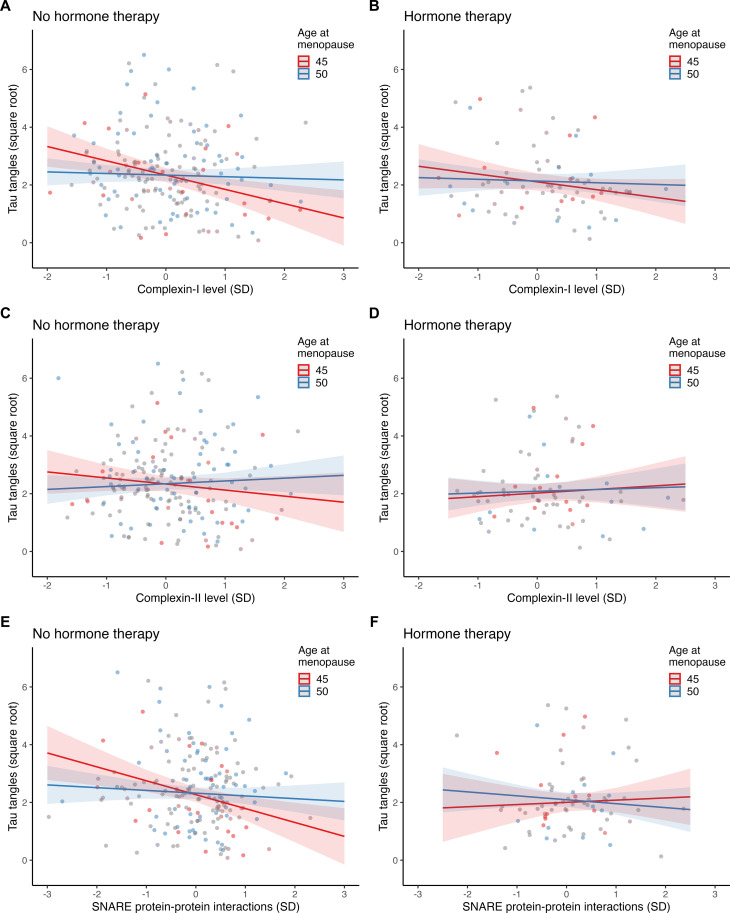
Synergistic associations of age at menopause and synaptic biomarkers on tau tangles stratified by history of menopausal hormone therapy. The plots depict separate models of the two-way interactions between age at menopause and levels of (**A** and **B**) complexin-1, (**C** and **D**) complexin-II, and (**E** and **F**) SNARE protein-protein interactions on tau tangles, in women with no history of hormone therapy (HT; left panels) and women with history of HT (right panels). The models are adjusted for age at death, years of education, β amyloid, *APOE* ε4 carriage, and mean SNARE density [for (C) only]. Age at menopause is modeled continuously, and model estimates for earlier (i.e., age 45) and average (i.e., age 50) ages at menopause are shown for visualization purposes. Shaded regions represent 95% CIs.

**Fig. 5.**
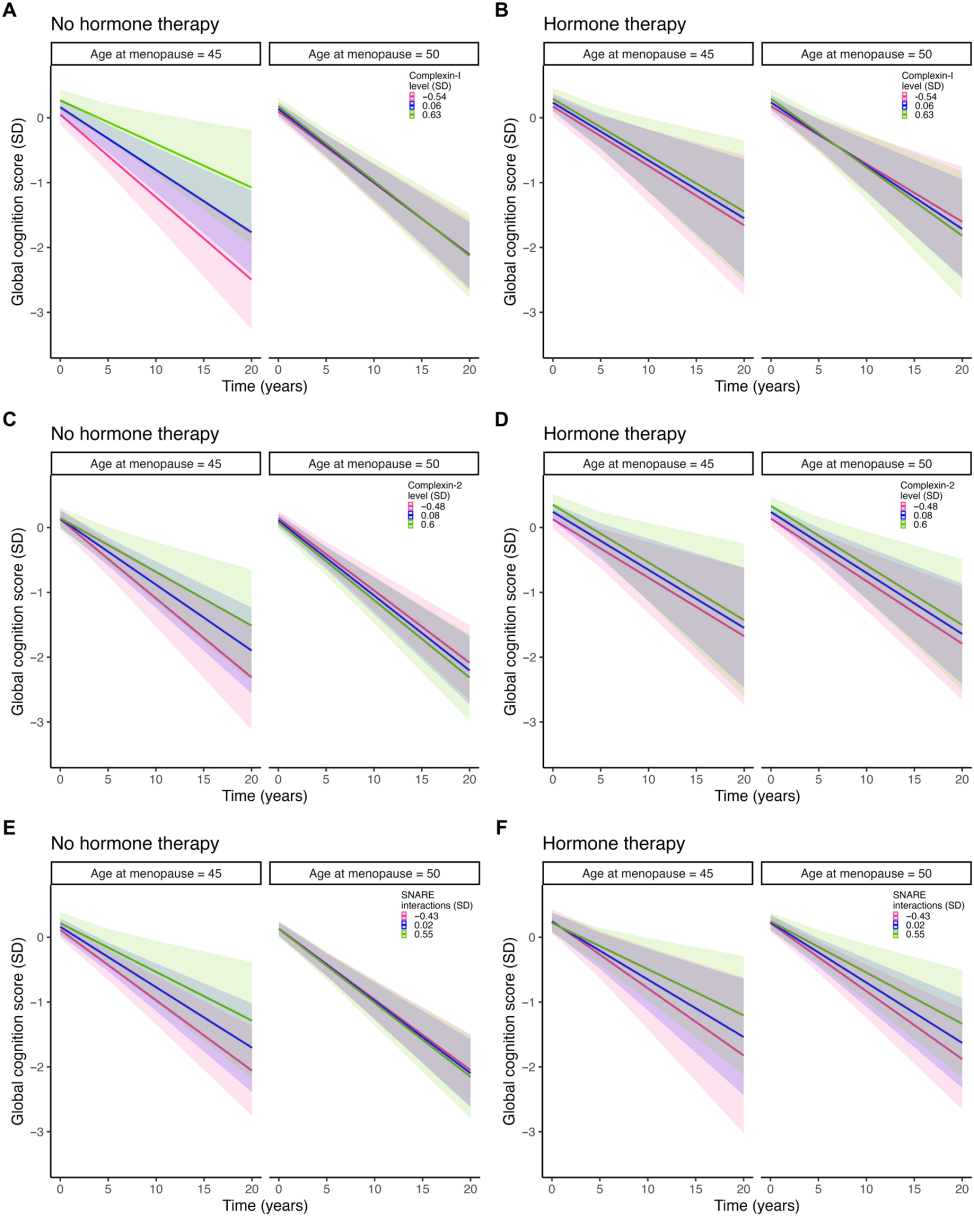
Synergistic associations of age at menopause and synaptic biomarkers on cognitive decline stratified by history of menopausal hormone therapy. The plots depict separate models of the three-way interactions between age at menopause; levels of (**A** and **B**) complexin-1, (**C** and **D**) complexin-II, and (**E** and **F**) SNARE protein-protein interactions; and time on global cognitive scores in women with no history of menopausal hormone therapy (HT; left panels) and women with history of HT (right panels). The models are adjusted for the interaction between age at death and time, along with years of education, *APOE* ε4 carriage, mean SNARE density [for (C) only], and their interactions with time. Age at menopause and synaptic marker levels are both modelled continuously. For visualization purposes, model estimates are shown for earlier (i.e., age 45) and average (i.e., age 50) ages at menopause and for the 25th, mean, and 75th percentiles of each synaptic marker. Shaded regions represent 95% CIs.

We next evaluated interactions between age at menopause and synaptic biomarkers on tau tangles and global cognitive decline in models stratified by lifetime history of hormone therapy (yes versus no). Similar to the primary analyses, women with no history of hormone therapy exhibited significant interactions between age at menopause and synaptic markers on tau tangles (table S2), as well as between age at menopause, synaptic markers, and time on cognitive decline (table S3). These models show that the relationships between synaptic dysfunction and greater tau or cognitive decline were stronger at earlier ages at menopause, among women with no history of hormone therapy (Figs. 4 and 5). These relationships were observed across all three synaptic biomarkers. By contrast, women who reported a history of hormone therapy use did not show significant interactions between age of menopause and synaptic biomarkers on tau (table S2) or cognitive outcomes (table S3) (Figs. 4 and 5). These findings suggest that hormone therapy may mitigate the observed negative effects of earlier age at menopause on synapse-related tau tangles and cognitive decline.

### Sensitivity analyses

In sensitivity analyses, we re-ran the main models adjusting for common non-AD pathologies, such as cerebral amyloid angiopathy, Lewy body pathology, TDP-43, hippocampal sclerosis, and vascular pathology. These models yielded similar results as those reported above (table S4).

## DISCUSSION

This longitudinal study of a deeply phenotyped cohort of women examined the interplay between age at menopause and biomarkers of synaptic integrity on post-mortem measures of AD pathology and longitudinal cognitive decline. We found that earlier age at menopause strengthened the effects of reduced synaptic integrity on cognitive decline, with greater levels of tau tangles mediating this association. The effects were specific to tau tangles, as no consistent or statistically significant associations were observed for β amyloid. These findings align with and extend the growing body of evidence that female-specific vulnerability to tau (rather than β amyloid) may underlie elevated AD risk in women (*4*, *15*, *29*, *30*). Exploratory analyses further suggested that associations of reduced synaptic integrity with greater tau tangles and faster cognitive decline were attenuated in women with a history of menopausal hormone therapy use. Together, these findings suggest that factors related to the menopause transition may play a key role in modulating synaptic contributions to AD risk in women.

Although interactive associations between age at menopause and synaptic integrity were observed across all three synaptic biomarkers when cognitive decline was the outcome (i.e., complexin-I, complexin-II, and SNARE protein–protein interactions), the pattern differed when tau was the outcome. In analyses examining tau tangles, only complexin-I and SNARE protein–protein interactions (but not complexin-II) showed interactive effects with age at menopause. This pattern aligns with previous findings in MAP specifically linking lower levels of complexin-I and SNARE protein–protein interactions (but not complexin-II) to greater tau in the setting of elevated β amyloid (*26*). Of note, while complexin-I is most prominently expressed on inhibitory neurons, complexin-II is more highly expressed on excitatory neurons. Extant literature implicates selective loss of inhibitory neurons in both aging and AD (*31*–*34*). Our results reinforce and build on these findings by providing evidence that dysfunction in inhibitory synaptic processes (i.e., complexin-I) is more strongly linked to tau than is dysfunction in excitatory synaptic processes (i.e., complexin-II). Further, our analyses implicate a menopause-related role for SNARE proteins, which facilitate vesicle trafficking and fusion at the presynaptic membrane to play a critical role in neurotransmitter release. The observed synergistic association between age at menopause and complexin-II on cognitive outcomes suggests that complexin-II may contribute to cognitive decline independently from tau. While the mechanisms remain unclear, lower levels of complexin-II (but not complexin-I) have been shown to associate with autopsy-assessed cortical atrophy, a link that may be mediated by other non-AD processes (*27*). Furthermore, the preserved integrity of excitatory neurons associated with complexin-II may contribute to cognitive reserve, independently from AD pathology (*27*). Together, our data suggest that the links between AD outcomes and presynaptic processes involving inhibitory neurons and neurotransmitter machinery may be sensitive to midlife endocrine processes or their downstream effects.

The potential mechanisms underlying the connections between age at menopause, synaptic biomarkers, and AD risk remain unclear. Estradiol, the most potent form of estrogen, exerts widespread beneficial effects on brain structure and function (*35*). A substantial body of research in humans suggests that the increased risk of AD associated with earlier menopause may be at least in part due to the loss of estradiol’s neuroprotective properties (*9*–*11*, *36*). Earlier menopause extends the period of estradiol deprivation, potentially increasing the brain’s susceptibility to AD pathology (*37*, *38*); estrogen depletion promotes increased tau hyperphosphorylation and reduced autophagy (*39*, *40*). Reduced synaptic integrity may exacerbate this vulnerability in women with earlier menopause. This hypothesis is further supported by the known interactions between estradiol and synaptic function (*28*), the loss of which may drive metabolic changes that lay the foundation for later AD pathogenesis (*11*, *12*). Another possibility is that earlier menopause represents a broader underlying vulnerability to aging processes. Earlier menopause accelerates biological aging as measured through epigenetic changes (*41*) and is associated with an increased risk of not only dementia but also a range of age-related and multi-organ conditions including cardiovascular diseases, osteoporosis, depression, and overall mortality (*42*–*45*). Thus, both earlier menopause and AD risk could result from shared upstream factors driving both outcomes. One possibility is genetic factors that influence inflammation, oxidative stress, and genome stability (*46*, *47*). Synergistic effects of age at menopause and synaptic integrity on AD risk may therefore reflect interactions between synaptic health and these upstream factors. Further, the specificity of these interactive effects—such that earlier menopause and reduced synaptic integrity relate to greater tau but not β amyloid—raise the possibility that tau is selectively influenced by sex-specific endocrine and/or other menopause-related processes. This idea is supported by in vivo evidence that menopause status moderates sex differences in tau (but not β amyloid) (*29*) and that women with younger age at menopause show greater tau burdens in the setting of elevated β amyloid (*15*). Future research should seek to clarify the role of estrogens and other key menopause-related biological changes in AD progression in women and shed light on how they relate to female tau vulnerability.

Contrary to previous findings in the MAP cohort (*23*, *26*, *27*, *31*), we did not observe statistically significant independent associations between synaptic biomarkers and either tau or cognitive decline. However, for tau analyses, effect sizes and directions were generally consistent with previous research showing greater tau burden at lower levels of synaptic biomarkers (*26*). By contrast, for analyses testing main associations of synaptic biomarkers with global cognitive decline, effect sizes were quite small, and directionality was inconsistent. These discrepancies might be due to the smaller sample size in our study, which focused on female participants with spontaneous menopause, and the generally small effects associated with these single analyte biomarkers. Another contributing factor may be that the relationships between synaptic biomarkers, tau, and cognitive decline in women differ by age at menopause, as suggested by our findings. Because the associations of synaptic dysfunction with greater tau and steeper cognitive decline were specific to women with earlier age at menopause, it is perhaps expected that these effects were nonsignificant when modeled in women across all ages at menopause and do not align with previous estimates from mixed-sex analyses.

We also observed no evidence of independent relationships between age at menopause and either synaptic biomarkers or AD outcomes (i.e., β amyloid, tau, and cognitive decline). Previous research has reported links between earlier age at menopause and elevated AD risk in other datasets (*15*, *17*, *18*). However, a prior study that used MAP data also found no associations of age at menopause with AD neuropathology or cognitive decline among women with spontaneous menopause (*16*). Notably, the average age at spontaneous menopause among white women in developed countries is typically reported to be within the range of 50 to 52 years (*46*), which is slightly older than mean ages at menopause reported in this study. While this discrepancy may relate to differing definitions of age at menopause among studies and/or secular trends in age at menopause (i.e., increasing age at menopause over time) (*46*, *48*), it may also reflect recruitment bias leading to biological idiosyncrasies among women participating in MAP. Hence, unmeasured demographic differences between study samples may contribute to inconsistent findings regarding the effects of age at menopause on AD outcomes.

Post hoc moderated mediation analyses suggested that the synergistic effects of age at menopause and synaptic dysfunction on cognitive decline were partially explained by higher levels of tau in women with both earlier menopause and reduced synaptic integrity. We frame these findings within the synaptic hypothesis of AD, which posits that synaptic dysregulation is an early phenomenon before fulminant tau tangles occur and causally contributes to AD pathogenesis (*49*). Preclinical research has found that disruptions in synaptic function may precede the development of AD pathology and further the formation and spread of β amyloid plaques and tau neurofibrillary tangles (*20*, *50*–*53*). However, we acknowledge that there is also evidence for reverse causality and bidirectionality in the associations between synaptic dysfunction and AD pathology (*54*, *55*). Moreover, preserved synaptic integrity may confer cognitive reserve and resilience independently from the effects of AD pathology (*24*, *25*, *27*). It is important to emphasize that our observational design cannot establish temporality or causality in the interplay between synaptic biomarkers, tau, and cognitive decline, and future experimental and in vivo research is needed to clarify these pathways.

Exploratory analyses showed that the interactive associations of age at menopause and synaptic markers on cognitive decline and tau tangles were largely attenuated in women with a history of menopausal hormone therapy. These findings align with previous research reporting that hormone therapy use may weaken the negative effects of earlier menopause on cognitive and AD outcomes (*17*, *19*, *56*). However, it is crucial to acknowledge that the data on hormone therapy were observational and retrospective in nature, which may introduce selection bias and residual confounding. Relevant to this possibility, we did observe several key demographic and clinical differences between women with versus without history of hormone therapy. Specifically, women with no hormone therapy were older at both baseline and death, had more tau tangles, and were more likely to be diagnosed with AD dementia. While the magnitudes of these differences were generally small, it is possible that the greater disease burden in the no hormone therapy group and/or other related but unmeasured demographic differences (e.g., access to health care) could have contributed to the differential synergistic effects of age at menopause and synaptic biomarkers between groups. Further, there is evidence that both the type (i.e., formulations and combinations of estrogens and progesterone) and timing of hormone therapy use may influence brain health outcomes (*15*, *19*, *57*–*59*). Unfortunately, no data were available on medication formulations, and a substantial proportion of participants had missing information on ages at HT initiation/cessation. As a result, we were unable to perform analyses based on hormone therapy type or timing/duration. Given these limitations, the exploratory findings on hormone therapy should be interpreted with caution.

There are several other important limitations. First, because all synaptic and neuropathological measures were assessed postmortem, we could not investigate temporal or causal dynamics of the associations between midlife endocrine factors, synaptic integrity, AD neuropathology, and cognitive decline. Future studies should use longitudinal in vivo measures of synaptic health (e.g., cerebrospinal fluid markers, positron emission tomography neuroimaging) and objective measures of hormonal transitions to clarify the connections between these factors. Second, as noted above, we relied on retrospectively self-reported data on menopause and hormone therapy history. Because no data were collected on oophorectomy among women with self-reported surgical menopause, we restricted our analyses to women with spontaneous menopause. Therefore, our findings may not generalize to women with hysterectomy and/or oophorectomy. Last, participants were well-educated, and the vast majority self-reported their race/ethnicity as white. This potentially limits the generalizability of our findings and underscores the critical need for greater diversity in dementia research.

To conclude, this study showed that women with earlier age at menopause exhibited stronger synaptic-related vulnerability to tau tangles and cognitive decline. These associations were less pronounced in women with a history of hormone therapy. Together, these results suggest that midlife endocrine processes and synaptic health may contribute to the well-established female-specific vulnerability to tau and the higher rates of AD dementia in women compared to men. Interventions targeting synaptic health could buffer menopause-related dementia risk to promote cognitive and neuropathological resilience to AD in women.

## MATERIALS AND METHODS

### Study design and participants

The objective of this study was to investigate the synergism between synaptic integrity and age at menopause on AD neuropathology and cognitive decline in women. Specifically, we examined whether age at spontaneous menopause modifies the associations of synaptic biomarkers with cognitive decline and post-mortem measures of AD pathology (i.e., tau tangles and β amyloid). An exploratory objective was to examine whether these effects differed between women with versus without a history of menopausal hormone therapy.

To address these aims, we used neuropathological and clinical data from the Rush MAP (*60*). MAP started in 1997 and enrolls community-dwelling participants across Northeastern Illinois. All participants have annual comprehensive clinical and cognitive evaluations and consent to brain donation. Participants undergo postmortem neuropathological evaluation, which includes quantification of presynaptic proteins in a subset of participants (*N* = 633) (*27*). MAP is approved by a Rush University Medical Center Institutional Review Board and conducted according to the latest Declaration of Helsinki. All participants provide written informed and repository consents and an Anatomic Gift Act.

In MAP, sex is self-reported as either female or male. No data were collected on sex at birth versus gender identity. Given that sex and gender are conflated and acknowledging that we cannot disentangle biological sex effects from those of sociocultural gender (*61*), we herein refer to female participants as “women” and male participants as “men.”

Analyses included women who self-reported spontaneous menopause and had complete synaptic biomarker, AD pathology, age at menopause, and covariate data (*N* = 268). Analyses of longitudinal cognitive decline additionally restricted the sample to participants with nonmissing cognitive data for at least two study visits (*N* = 253). The sample selection process is detailed in fig. S3. We followed the Strengthening the Reporting of Observational Studies in Epidemiology guidelines for cohort studies.

### Synaptic biomarkers

For neuropathological evaluation, brains from decedents were removed and prepared following previously described protocols (*62*). Presynaptic proteins were assessed using frozen gray matter samples from six brain regions (i.e., hippocampus, middle frontal gyrus, inferior temporal gyrus, calcarine cortex, ventromedial caudate, and posterior putamen). As previously described (*27*), monoclonal antibodies were used to measure immunoreactivity for each protein using enzyme-linked immunosorbent assay (ELISA). Assayed proteins included complexin-I (inhibitory), complexin-II (excitatory), and three SNARE proteins (SNAP-25, VAMP, and syntaxin-1). For each protein, regional immunodensity values were log-transformed, standardized, and averaged across brain regions to produce a composite score of overall presynaptic protein integrity for each participant. Standardized scores were multiplied by negative 1, such that higher values correspond to greater protein densities (i.e., greater synaptic integrity). Protein-protein interactions between SNARE proteins (i.e., SNAP-25, VAMP, and syntaxin-1) were also quantified via a high-throughput immunoprecipitation strategy using a heterologous capture ELISA (*27*, *63*). As for the protein density measures, values for each pair of interacting proteins were log-transformed, standardized, averaged across brain regions, and multiplied by negative 1. Then, a mean score for SNARE protein-protein interactions was computed by averaging the composite scores for each of the four measured protein-protein interactions. Higher values reflect greater functional capacity of the presynaptic terminals to release neurotransmitters.

In the present study, we examined presynaptic terminal integrity via average levels of complexin-I and complexin-II and presynaptic terminal functionality via average SNARE protein-protein interactions. These markers were selected as they have previously been shown to be associated with AD neuropathology and cognitive decline in MAP (*22*, *23*, *26*, *27*). To reduce multiple comparisons, we chose to focus on regionally global composite scores for each synaptic biomarker examined.

### Alzheimer’s disease neuropathology

As previously described (*64*), β amyloid and tau tangles were quantified using molecularly specific immunohistochemistry in eight brain regions: the hippocampus, entorhinal cortex, midfrontal cortex, inferior temporal cortex, angular gyrus, calcarine cortex, anterior cingulate cortex, and superior frontal cortex. For each region, levels of β amyloid (percentage area occupied) and tau (cortical density per square millimeter) were calculated using image analysis. Composite scores for each β amyloid and tau tangles were produced by averaging the values for each region. As previously done, summary measures of β amyloid and tau tangles were square root–transformed before model entry due to their skewed distributions (*65*). Findings of other common neuropathologies including cerebral amyloid angiopathy (CAA), vascular pathology (i.e., macroinfarcts, microinfarcts, arteriosclerosis, and atherosclerosis), Lewy body disease, TAR DNA binding protein 43 (TDP-43), and hippocampal sclerosis were also quantified, as previously described (*62*).

### Women’s reproductive health history

At study baseline, women self-reported their age at menopause (“At what age did you stop menstruating?”) and whether menopause was “natural” (i.e., spontaneous) or “caused by surgery.” No data were collected on whether surgical menopause included oophorectomy, so reported age at menstrual cessation may not accurately reflect age at ovarian failure in women with surgical menopause (*66*). Hence, analyses only included women with spontaneous menopause (*N* = 143 with surgical or unknown cause of menopause excluded). As previously done (*15*, *16*), we excluded women who reported improbable ages at spontaneous menopause (i.e., <20 or >60 years; *N* = 5).

History of menopausal hormone therapy was also self-reported at study baseline. Women were asked whether they have ever taken medication for hormone replacement, and if they responded affirmatively, and were asked to identify route of administration (i.e., pill, injection, vaginal cream or suppository, or skin patch). Participants were further asked to report the ages at which they started and stopped taking hormone therapy. The total duration of hormone therapy use was calculated by subtracting age at initiation from age at cessation.

Exploratory hormone therapy analyses compared women who reported ever taking hormone therapy to those who reported never taking hormone therapy. These analyses excluded *N* = 1 participant with missing data on lifetime hormone therapy use and *N* = 3 participants with “suspect” hormone therapy use (i.e., reflecting an inconclusive or uncertain response to the question of lifetime hormone therapy use). These exclusions resulted in an analytic sample of *N* = 264 for exploratory hormone therapy analyses (*N* = 75 users and *N* = 189 non-users). Analyses of longitudinal cognitive decline additionally restricted the hormone therapy sample to participants with nonmissing cognitive data for at least two study visits (*N* = 250; including *N* = 71 users and *N* = 179 non-users). A substantial proportion of participants who reported taking hormone therapy were missing data on age at initiation/cessation and duration (*N* = 25, 33.3%), so we did not make further exclusions or perform analyses based on hormone therapy timing or duration.

### Cognition

Cognition was assessed approximately annually with a battery of neuropsychological tests (*60*). The battery assesses a broad spectrum of cognitive functions, including episodic memory, semantic memory, working memory, perceptual speed, and visuospatial ability. We focused on a composite score of global cognition, which has previously demonstrated associations with synaptic biomarkers (*23*). The global cognitive composite was computed by averaging standardized test scores for 19 tests in the battery (*67*).

Cognitive and clinical status is assessed annually. Diagnoses of mild cognitive impairment (MCI), AD, and other dementias are made based on a three-stage process that includes scoring of neuropsychological tests, clinical assessment by a neuropsychologist, and diagnostic classification by a clinician (*68*).

### *APOE* genotype

The single-nucleotide polymorphisms rs429358 and rs7412 were used to determine apolipoprotein E (*APOE*) genotype from DNA extracted from peripheral blood or frozen post-mortem brain tissue (*69*). We categorized participants with one or two copies of *APOE* ε4 as ε4 carriers and remaining participants as noncarriers.

### Statistical analysis

Analyses were conducted in R (v.4.4.1). We calculated descriptive statistics for demographic and clinical variables for both the main analytic sample and exploratory hormone therapy subsample. We evaluated data distributions for normality using visual inspection of histograms and Q-Q plots as well as Shapiro-Wilk tests. We used Welch’s unequal variance *t* tests (where the assumption of normality was met), Wilcoxon rank sum tests (where data distributions deviated from normality), and χ^2^ tests to assess differences between women with versus without history of hormone therapy.

Results with *P* < 0.05 were considered statistically significant. Despite the number of models evaluated, we did not adjust for multiple comparisons and instead focused on the biological coherence of the findings, which emphasizes the effect size, directionality, and consistency of relationships, with less emphasis on statistical significance, per recommendations of the American Statistical Association (*70*). For all models, synaptic biomarkers and age at menopause were entered as continuous variables. For visualization purposes, we show model estimates for ages at menopause of 45 years [representing the clinical cutoff for early menopause (*71*)] and 50 years [representing the approximate average age at spontaneous menopause in the United States (*48*)]. Where appropriate, we also show model estimates for low (i.e., 25th percentile), average (i.e., mean), and high (i.e., 75th percentile) levels of synaptic biomarkers.

#### Neuropathology analyses

We first used linear models to test direct associations between age at menopause, each synaptic biomarker (i.e., complexin-I, complexin-II, and SNARE protein-protein interactions), and AD neuropathology (i.e., tau tangles and β amyloid). To evaluate whether age at menopause modifies the associations of synaptic biomarkers with AD neuropathology, a series of separate linear models tested the interactions between each synaptic and age at menopause on tau tangles and β amyloid. Models were adjusted for age at death, years of education, and *APOE* ε4 carriage. Models where SNARE protein-protein interactions were a predictor additionally adjusted for mean SNARE protein density. Models where tau was an outcome also adjusted for β amyloid.

#### Cognitive analyses

Cognitive analyses consisted of a series of linear mixed effects models evaluating change in global cognition scores over time. All models included random slopes and intercepts and adjusted for years of education, *APOE* ε4 carriage, and their interactions with time. To control for both baseline age and age at death, which are inherently collinear, we adjusted for the interaction between baseline age and number of visits, as previously done (*72*). Models where SNARE protein-protein interactions were a predictor additionally adjusted for mean SNARE protein density and its interaction with time.

To test main associations between key variables of interest, we first evaluated separate models testing the interaction of each synaptic biomarker (i.e., complexin-I, complexin-II, and SNARE protein-protein interactions) and time on cognitive scores, as well as the interaction of age at menopause and time on cognitive scores. Next, to investigate whether age at menopause modifies the associations of synaptic biomarkers with cognitive decline, we modeled the three-way interactions between each synaptic biomarker, age at menopause, and time on cognitive scores.

#### Post hoc mediation analyses

Because we observed that earlier menopause exacerbated the associations of synaptic biomarkers (i.e., complexin-I and SNARE protein-protein interactions) with tau tangles and global cognitive decline and given the evidence that synaptic dysregulation may causally contribute to AD pathogenesis (*20*, *37*, *49*–*52*), we tested whether the interactions between synaptic biomarkers and age at menopause on cognitive decline were mediated by tau levels in post hoc analyses. We first extracted slopes of cognitive change from an unadjusted linear mixed effects model. Then, we performed moderated mediation analyses using the “mediation” package in R. For each mediation analysis, we estimated two regression models. First, we modeled the interaction between the synaptic biomarker and age at menopause on tau tangles, adjusting for age at death, years of education, β amyloid, *APOE* ε4, and mean SNARE density (for SNARE protein-protein interactions model only). We then evaluated a separate model with two predictors: (i) the interaction between the synaptic biomarker and age at menopause and (ii) tau, with cognitive slope as the outcome. This second model adjusted for the interaction between age at baseline and number of visits, years of education, β amyloid, *APOE* ε4, and mean SNARE density (for SNARE protein-protein interactions model only). We performed mediation analyses at two levels of age at menopause (i.e., the moderator): age 45 (representing earlier menopause) and age 50 (representing average menopause). We estimated the ACMEs and the ADEs using nonparametric bootstrapping with 1000 simulations.

#### Exploratory menopausal hormone therapy analyses

We lastly explored whether the synergistic effects of synaptic biomarkers and age at menopause on AD neuropathology and cognitive decline differed by history of hormone therapy. Given the relatively small sample sizes and to avoid modeling four-way interactions (i.e., in cognitive analyses), to address this exploratory aim, we repeated the above models stratified by hormone therapy history (i.e., lifetime use versus non-use). Models adjusted for the same covariates detailed above. Effect sizes and significance levels were compared to infer differences by history of hormone therapy.

#### Sensitivity analyses

In sensitivity analyses, we re-evaluated main models additionally adjusting for common non-AD neuropathologies, including CAA (rated as none, mild, moderate, or severe), Lewy body disease (dichotomized as absent/nigral-predominant versus limbic/neocortical-type), TDP-43 (dichotomized as absent/amygdala-only versus limbic/neocortical), hippocampal sclerosis (absent versus present), and vascular pathology [quantified with a summary score of the presence/absence and severity of macroinfarcts, microinfarcts, arteriosclerosis, and atherosclerosis, as previously done (*73*)].

## References

[1] M. T. Ferretti, M. F. Iulita, E. Cavedo, P. A. Chiesa, A. Schumacher Dimech, A. Santuccione Chadha, F. Baracchi, H. Girouard, S. Misoch, E. Giacobini, H. Depypere, H. Hampel, Women’s Brain Project and the Alzheimer Precision Medicine Initiative, Sex differences in Alzheimer disease—The gateway to precision medicine. Nat. Rev. Neurol. 14, 457–469 (2018). 29985474 10.1038/s41582-018-0032-9

[2] G. Chêne, A. Beiser, R. Au, S. R. Preis, P. A. Wolf, C. Dufouil, S. Seshadri, Gender and incidence of dementia in the Framingham Heart Study from mid-adult life. Alzheimers Dement. 11, 310–320 (2015). 24418058 10.1016/j.jalz.2013.10.005 PMC4092061

[3] R. A. Nebel, N. T. Aggarwal, L. L. Barnes, A. Gallagher, J. M. Goldstein, K. Kantarci, M. P. Mallampalli, E. C. Mormino, L. Scott, W. H. Yu, P. M. Maki, M. M. Mielke, Understanding the impact of sex and gender in Alzheimer’s disease: A call to action. Alzheimers Dement. 14, 1171–1183 (2018). 29907423 10.1016/j.jalz.2018.04.008 PMC6400070

[4] R. F. Buckley, E. C. Mormino, J. S. Rabin, T. J. Hohman, S. Landau, B. J. Hanseeuw, H. I. L. Jacobs, K. V. Papp, R. E. Amariglio, M. J. Properzi, A. P. Schultz, D. Kirn, M. R. Scott, T. Hedden, M. Farrell, J. Price, J. Chhatwal, D. M. Rentz, V. L. Villemagne, K. A. Johnson, R. A. Sperling, Sex differences in the association of global amyloid and regional tau deposition measured by positron emission tomography in clinically normal older adults. JAMA Neurol. 76, 542–551 (2019). 30715078 10.1001/jamaneurol.2018.4693 PMC6515599

[5] T. J. Hohman, L. Dumitrescu, L. L. Barnes, M. Thambisetty, G. Beecham, B. Kunkle, K. A. Gifford, W. S. Bush, L. B. Chibnik, S. Mukherjee, P. L. De Jager, W. Kukull, P. K. Crane, S. M. Resnick, C. D. Keene, T. J. Montine, G. D. Schellenberg, J. L. Haines, H. Zetterberg, K. Blennow, E. B. Larson, S. C. Johnson, M. Albert, D. A. Bennett, J. A. Schneider, A. L. Jefferson, Alzheimer’s Disease Genetics Consortium and the Alzheimer’s Disease Neuroimaging Initiative, Sex-specific association of apolipoprotein e with cerebrospinal fluid levels of Tau. JAMA Neurol. 75, 989–998 (2018). 29801024 10.1001/jamaneurol.2018.0821 PMC6142927

[6] R. F. Buckley, E. C. Mormino, R. E. Amariglio, M. J. Properzi, J. S. Rabin, Y. Y. Lim, K. V. Papp, H. I. L. Jacobs, S. Burnham, B. J. Hanseeuw, V. Doré, A. Dobson, C. L. Masters, M. Waller, C. C. Rowe, P. Maruff, M. C. Donohue, D. M. Rentz, D. Kirn, T. Hedden, J. Chhatwal, A. P. Schultz, K. A. Johnson, V. L. Villemagne, R. A. Sperling, Alzheimer's Disease Neuroimaging Initiative, Australian Imaging, Biomarker and Lifestyle study of ageing, Harvard Aging Brain Study, Sex, amyloid, and APOE ε4 and risk of cognitive decline in preclinical Alzheimer’s disease: Findings from three well-characterized cohorts. Alzheimers Dement. 14, 1193–1203 (2018). 29803541 10.1016/j.jalz.2018.04.010 PMC6131023

[7] M. E. I. Koran, M. Wagener, T. J. Hohman, Alzheimer’s Neuroimaging Initiative, Sex Differences in the association between AD biomarkers and cognitive decline. Brain Imaging Behav. 11, 205–213 (2017). 26843008 10.1007/s11682-016-9523-8 PMC4972701

[8] M.-A. Arevalo, I. Azcoitia, L. M. Garcia-Segura, The neuroprotective actions of oestradiol and oestrogen receptors. Nat. Rev. Neurosci. 16, 17–29 (2015). 25423896 10.1038/nrn3856

[9] S. Jett, E. Schelbaum, G. Jang, C. Boneu Yepez, J. P. Dyke, S. Pahlajani, R. Diaz Brinton, L. Mosconi, Ovarian steroid hormones: A long overlooked but critical contributor to brain aging and Alzheimer’s disease. Front. Aging Neurosci. 14, 948219 (2022). 35928995 10.3389/fnagi.2022.948219 PMC9344010

[10] C. J. Pike, J. C. Carroll, E. R. Rosario, A. Barron, Protective actions of sex steroid hormones in Alzheimer’s disease. Front. Neuroendocrinol. 30, 239–258 (2009). 19427328 10.1016/j.yfrne.2009.04.015 PMC2728624

[11] R. D. Brinton, J. Yao, F. Yin, W. J. Mack, E. Cadenas, Perimenopause as a neurological transition state. Nat. Rev. Endocrinol. 11, 393–405 (2015). 26007613 10.1038/nrendo.2015.82 PMC9934205

[12] F. Yin, J. Yao, H. Sancheti, T. Feng, R. C. Melcangi, T. E. Morgan, C. E. Finch, C. J. Pike, W. J. Mack, E. Cadenas, R. D. Brinton, The perimenopausal aging transition in the female rat brain: Decline in bioenergetic systems and synaptic plasticity. Neurobiol. Aging 36, 2282–2295 (2015). 25921624 10.1016/j.neurobiolaging.2015.03.013 PMC4416218

[13] M. Nerattini, F. Rubino, S. Jett, C. Andy, C. Boneu, C. Zarate, C. Carlton, S. Loeb-Zeitlin, Y. Havryliuk, S. Pahlajani, S. Williams, V. Berti, P. Christos, M. Fink, J. P. Dyke, R. D. Brinton, L. Mosconi, Elevated gonadotropin levels are associated with increased biomarker risk of Alzheimer’s disease in midlife women. Front. Dement. 2, 1303256 (2023). 38774256 10.3389/frdem.2023.1303256 PMC11108587

[14] J. Xiong, S. S. Kang, M. Wang, Z. Wang, Y. Xia, J. Liao, X. Liu, S.-P. Yu, Z. Zhang, V. Ryu, T. Yuen, M. Zaidi, K. Ye, FSH and ApoE4 contribute to Alzheimer’s disease-like pathogenesis via C/EBPβ/δ-secretase in female mice. Nat. Commun. 14, 6577 (2023). 37852961 10.1038/s41467-023-42282-7 PMC10584868

[15] G. T. Coughlan, T. J. Betthauser, R. Boyle, R. L. Koscik, H. M. Klinger, L. B. Chibnik, E. M. Jonaitis, W.-Y. W. Yau, A. Wenzel, B. T. Christian, C. E. Gleason, U. G. Saelzler, M. J. Properzi, A. P. Schultz, B. J. Hanseeuw, J. E. Manson, D. M. Rentz, K. A. Johnson, R. Sperling, S. C. Johnson, R. F. Buckley, Association of age at menopause and hormone therapy use with Tau and β-amyloid positron emission tomography. JAMA Neurol. 80, 462 (2023). 37010830 10.1001/jamaneurol.2023.0455 PMC10071399

[16] R. Bove, E. Secor, L. B. Chibnik, L. L. Barnes, J. A. Schneider, D. A. Bennett, P. L. De Jager, Age at surgical menopause influences cognitive decline and Alzheimer pathology in older women. Neurology 82, 222–229 (2014). 24336141 10.1212/WNL.0000000000000033 PMC3902759

[17] M. Wood Alexander, C.-Y. Wu, G. T. Coughlan, T. Puri, R. F. Buckley, P. Palta, W. Swardfager, M. Masellis, L. A. M. Galea, G. Einstein, S. E. Black, J. S. Rabin, Associations between age at menopause, vascular risk, and 3-year cognitive change in the Canadian Longitudinal Study on aging. Neurology 102, e209298 (2024). 38569140 10.1212/WNL.0000000000209298

[18] J. Gong, K. Harris, S. A. E. Peters, M. Woodward, Reproductive factors and the risk of incident dementia: A cohort study of UK Biobank participants. PLOS Med. 19, e1003955 (2022). 35381014 10.1371/journal.pmed.1003955 PMC8982865

[19] M. Nerattini, S. Jett, C. Andy, C. Carlton, C. Zarate, C. Boneu, M. Battista, S. Pahlajani, S. Loeb-Zeitlin, Y. Havryulik, S. Williams, P. Christos, M. Fink, R. D. Brinton, L. Mosconi, Systematic review and meta-analysis of the effects of menopause hormone therapy on risk of Alzheimer’s disease and dementia. Front. Aging Neurosci. 15, 1260427 (2023). 37937120 10.3389/fnagi.2023.1260427 PMC10625913

[20] M. Tzioras, R. I. McGeachan, C. S. Durrant, T. L. Spires-Jones, Synaptic degeneration in Alzheimer disease. Nat. Rev. Neurol. 19, 19–38 (2023). 36513730 10.1038/s41582-022-00749-z

[21] A. P. Mecca, R. S. O’Dell, E. S. Sharp, E. R. Banks, H. H. Bartlett, W. Zhao, S. Lipior, N. G. Diepenbrock, M.-K. Chen, M. Naganawa, T. Toyonaga, N. B. Nabulsi, B. C. Vander Wyk, A. F. T. Arnsten, Y. Huang, R. E. Carson, C. H. van Dyck, Synaptic density and cognitive performance in Alzheimer’s disease: A PET imaging study with [^11^C]UCB-J. Alzheimers Dement. 18, 2527–2536 (2022). 35174954 10.1002/alz.12582 PMC9381645

[22] A. Ramos-Miguel, A. A. Jones, K. Sawada, A. M. Barr, T. A. Bayer, P. Falkai, S. E. Leurgans, J. A. Schneider, D. A. Bennett, W. G. Honer, Frontotemporal dysregulation of the SNARE protein interactome is associated with faster cognitive decline in old age. Neurobiol. Dis. 114, 31–44 (2018). 29496544 10.1016/j.nbd.2018.02.006 PMC6483375

[23] P. A. Boyle, R. S. Wilson, L. Yu, A. M. Barr, W. G. Honer, J. A. Schneider, D. A. Bennett, Much of late life cognitive decline is not due to common neurodegenerative pathologies. Ann. Neurol. 74, 478–489 (2013). 23798485 10.1002/ana.23964 PMC3845973

[24] S. E. Arnold, N. Louneva, K. Cao, L.-S. Wang, L.-Y. Han, D. A. Wolk, S. Negash, S. E. Leurgans, J. A. Schneider, A. S. Buchman, R. S. Wilson, D. A. Bennett, Cellular, synaptic, and biochemical features of resilient cognition in Alzheimer’s disease. Neurobiol. Aging 34, 157–168 (2013). 22554416 10.1016/j.neurobiolaging.2012.03.004 PMC3478410

[25] B. D. Boros, K. M. Greathouse, E. G. Gentry, K. A. Curtis, E. L. Birchall, M. Gearing, J. H. Herskowitz, Dendritic spines provide cognitive resilience against Alzheimer’s disease. Ann. Neurol. 82, 602–614 (2017). 28921611 10.1002/ana.25049 PMC5744899

[26] K. B. Casaletto, H. Zetterberg, K. Blennow, A. Brinkmalm, W. Honer, J. A. Schneider, D. A. Bennett, N. Djukic, M. You, S. Weiner-Light, C. Fonseca, B. L. Miller, J. Kramer, Tripartite relationship among synaptic, amyloid, and Tau proteins: An in vivo and postmortem study. Neurology 97, e284–e297 (2021). 33947778 10.1212/WNL.0000000000012145 PMC8302153

[27] W. G. Honer, A. M. Barr, K. Sawada, A. E. Thornton, M. C. Morris, S. E. Leurgans, J. A. Schneider, D. A. Bennett, Cognitive reserve, presynaptic proteins and dementia in the elderly. Transl. Psychiatry 2, e114 (2012). 22832958 10.1038/tp.2012.38 PMC3365257

[28] Y. Hara, E. M. Waters, B. S. McEwen, J. H. Morrison, Estrogen effects on cognitive and synaptic health over the lifecourse. Physiol. Rev. 95, 785–807 (2015). 26109339 10.1152/physrev.00036.2014 PMC4491541

[29] R. F. Buckley, A. O’Donnell, E. R. McGrath, H. I. L. Jacobs, C. Lois, C. L. Satizabal, S. Ghosh, Z. B. Rubinstein, J. M. Murabito, R. A. Sperling, K. A. Johnson, S. Seshadri, A. S. Beiser, Menopause status moderates sex differences in Tau burden: A Framingham PET study. Ann. Neurol. 92, 11–22 (2022). 35471588 10.1002/ana.26382 PMC9233144

[30] P. Duarte-Guterman, A. Y. Albert, C. K. Barha, L. A. M. Galea, on behalf of the Alzheimer’s Disease Neuroimaging Initiative, Sex influences the effects of APOE genotype and Alzheimer’s diagnosis on neuropathology and memory. Psychoneuroendocrinology 129, 105248 (2021). 33962245 10.1016/j.psyneuen.2021.105248

[31] W. G. Honer, A. Ramos-Miguel, J. Alamri, K. Sawada, A. M. Barr, J. A. Schneider, D. A. Bennett, The synaptic pathology of cognitive life. Dialogues Clin. Neurosci. 21, 271–279 (2019). 31749651 10.31887/DCNS.2019.21.3/whoner PMC6829169

[32] E. Vico Varela, G. Etter, S. Williams, Excitatory-inhibitory imbalance in Alzheimer’s disease and therapeutic significance. Neurobiol. Dis. 127, 605–615 (2019). 30999010 10.1016/j.nbd.2019.04.010

[33] H. Kurucu, M. Colom-Cadena, C. Davies, L. Wilkins, D. King, J. Rose, M. Tzioras, J. H. Tulloch, C. Smith, T. L. Spires-Jones, Inhibitory synapse loss and accumulation of amyloid beta in inhibitory presynaptic terminals in Alzheimer’s disease. Eur. J. Neurol. 29, 1311–1323 (2022). 34331352 10.1111/ene.15043

[34] J. C. Lauterborn, P. Scaduto, C. D. Cox, A. Schulmann, G. Lynch, C. M. Gall, C. D. Keene, A. Limon, Increased excitatory to inhibitory synaptic ratio in parietal cortex samples from individuals with Alzheimer’s disease. Nat. Commun. 12, 2603 (2021). 33972518 10.1038/s41467-021-22742-8 PMC8110554

[35] J. R. Rettberg, J. Yao, R. D. Brinton, Estrogen: A master regulator of bioenergetic systems in the brain and body. Front. Neuroendocrinol. 35, 8–30 (2014). 23994581 10.1016/j.yfrne.2013.08.001 PMC4024050

[36] S. Jett, N. Malviya, E. Schelbaum, G. Jang, E. Jahan, K. Clancy, H. Hristov, S. Pahlajani, K. Niotis, S. Loeb-Zeitlin, Y. Havryliuk, R. Isaacson, R. D. Brinton, L. Mosconi, Endogenous and exogenous estrogen exposures: How women’s reproductive health can drive brain aging and inform Alzheimer’s prevention. Front. Aging Neurosci. 14, 831807 (2022). 35356299 10.3389/fnagi.2022.831807 PMC8959926

[37] L. Mosconi, V. Berti, C. Guyara-Quinn, P. McHugh, G. Petrongolo, R. S. Osorio, C. Connaughty, A. Pupi, S. Vallabhajosula, R. S. Isaacson, M. J. de Leon, R. H. Swerdlow, R. D. Brinton, Perimenopause and emergence of an Alzheimer’s bioenergetic phenotype in brain and periphery. PLOS ONE 12, e0185926 (2017). 29016679 10.1371/journal.pone.0185926 PMC5634623

[38] Y. Wang, Y. Shang, A. Mishra, E. Bacon, F. Yin, R. Brinton, Midlife chronological and endocrinological transitions in brain metabolism: System biology basis for increased alzheimer’s risk in female brain. Sci. Rep. 10, 8528 (2020). 32444841 10.1038/s41598-020-65402-5 PMC7244485

[39] E. E. Congdon, Sex differences in autophagy contribute to female vulnerability in Alzheimer’s disease. Front. Neurosci. 12, 372 (2018). 29988365 10.3389/fnins.2018.00372 PMC6023994

[40] D. Muñoz-Mayorga, C. Guerra-Araiza, L. Torner, T. Morales, Tau phosphorylation in female neurodegeneration: Role of estrogens, progesterone, and prolactin. Front. Endocrinol. 9, 133 (2018). 10.3389/fendo.2018.00133 PMC5882780 29643836

[41] M. E. Levine, A. T. Lu, B. H. Chen, D. G. Hernandez, A. B. Singleton, L. Ferrucci, S. Bandinelli, E. Salfati, J. E. Manson, A. Quach, C. D. J. Kusters, D. Kuh, A. Wong, A. E. Teschendorff, M. Widschwendter, B. R. Ritz, D. Absher, T. L. Assimes, S. Horvath, Menopause accelerates biological aging. Proc. Natl. Acad. Sci. U.S.A. 113, 9327–9332 (2016). 27457926 10.1073/pnas.1604558113 PMC4995944

[42] T. Muka, C. Oliver-Williams, S. Kunutsor, J. S. E. Laven, B. C. J. M. Fauser, R. Chowdhury, M. Kavousi, O. H. Franco, Association of age at onset of menopause and time since onset of menopause with cardiovascular outcomes, intermediate vascular traits, and all-cause mortality: A systematic review and meta-analysis. JAMA Cardiol. 1, 767–776 (2016). 27627190 10.1001/jamacardio.2016.2415

[43] O. Svejme, H. Ahlborg, J.-Å. Nilsson, M. Karlsson, Early menopause and risk of osteoporosis, fracture and mortality: A 34-year prospective observational study in 390 women. BJOG 119, 810–816 (2012). 22531019 10.1111/j.1471-0528.2012.03324.x

[44] M. E. Ossewaarde, M. L. Bots, A. L. M. Verbeek, P. H. M. Peeters, Y. van der Graaf, D. E. Grobbee, Y. T. van der Schouw, Age at menopause, cause-specific mortality and total life expectancy. Epidemiology 16, 556–562 (2005). 15951675 10.1097/01.ede.0000165392.35273.d4

[45] M. K. Georgakis, T. P. Thomopoulos, A.-A. Diamantaras, E. I. Kalogirou, A. Skalkidou, S. S. Daskalopoulou, E. T. Petridou, Association of age at menopause and duration of reproductive period with depression after menopause: A systematic review and meta-analysis. JAMA Psychiatry 73, 139–149 (2016). 26747373 10.1001/jamapsychiatry.2015.2653

[46] E. B. Gold, The timing of the age at which natural menopause occurs. Obstet. Gynecol. Clin. North Am. 38, 425–440 (2011). 21961711 10.1016/j.ogc.2011.05.002 PMC3285482

[47] L. Stolk, J. R. Perry, D. I. Chasman, C. He, M. Mangino, P. Sulem, M. Barbalic, L. Broer, E. M. Byrne, F. Ernst, T. Esko, N. Franceschini, D. F. Gudbjartsson, J.-J. Hottenga, P. Kraft, P. F. McArdle, E. Porcu, S.-Y. Shin, A. V. Smith, S. van Wingerden, G. Zhai, W. V. Zhuang, E. Albrecht, B. Z. Alizadeh, T. Aspelund, S. Bandinelli, L. B. Lauc, J. S. Beckmann, M. Boban, E. Boerwinkle, F. J. Broekmans, A. Burri, H. Campbell, S. J. Chanock, C. Chen, M. C. Cornelis, T. Corre, A. D. Coviello, P. d’Adamo, G. Davies, U. de Faire, E. J. de Geus, I. J. Deary, G. V. Dedoussis, P. Deloukas, S. Ebrahim, G. Eiriksdottir, V. Emilsson, J. G. Eriksson, B. C. Fauser, L. Ferreli, L. Ferrucci, K. Fischer, A. R. Folsom, M. E. Garcia, P. Gasparini, C. Gieger, N. Glazer, D. E. Grobbee, P. Hall, T. Haller, S. E. Hankinson, M. Hass, C. Hayward, A. C. Heath, A. Hofman, E. Ingelsson, A. C. J. Janssens, A. D. Johnson, D. Karasik, S. L. Kardia, J. Keyzer, D. P. Kiel, I. Kolcic, Z. Kutalik, J. Lahti, S. Lai, T. Laisk, J. S. Laven, D. A. Lawlor, J. Liu, L. M. Lopez, Y. V. Louwers, P. K. Magnusson, M. Marongiu, N. G. Martin, I. M. Klaric, C. Masciullo, B. McKnight, S. E. Medland, D. Melzer, V. Mooser, P. Navarro, A. B. Newman, D. R. Nyholt, N. C. Onland-Moret, A. Palotie, G. Paré, A. N. Parker, N. L. Pedersen, P. H. Peeters, G. Pistis, A. S. Plump, O. Polasek, V. J. Pop, B. M. Psaty, K. Räikkönen, E. Rehnberg, J. I. Rotter, I. Rudan, C. Sala, A. Salumets, A. Scuteri, A. Singleton, J. A. Smith, H. Snieder, N. Soranzo, S. N. Stacey, J. M. Starr, M. G. Stathopoulou, K. Stirrups, R. P. Stolk, U. Styrkarsdottir, Y. V. Sun, A. Tenesa, B. Thorand, D. Toniolo, L. Tryggvadottir, K. Tsui, S. Ulivi, R. M. van Dam, Y. T. van der Schouw, C. H. van Gils, P. van Nierop, J. M. Vink, P. M. Visscher, M. Voorhuis, G. Waeber, H. Wallaschofski, H. E. Wichmann, E. Widen, C. J. W. Gent, G. Willemsen, J. F. Wilson, B. H. Wolffenbuttel, A. F. Wright, L. M. Yerges-Armstrong, T. Zemunik, L. Zgaga, M. C. Zillikens, M. Zygmunt, A. M. Arnold, D. I. Boomsma, J. E. Buring, L. Crisponi, E. W. Demerath, V. Gudnason, T. B. Harris, F. B. Hu, D. J. Hunter, L. J. Launer, A. Metspalu, G. W. Montgomery, B. A. Oostra, P. M. Ridker, S. Sanna, D. Schlessinger, T. D. Spector, K. Stefansson, E. A. Streeten, U. Thorsteinsdottir, M. Uda, A. G. Uitterlinden, C. M. van Duijn, H. Völzke, A. Murray, J. M. Murabito, J. A. Visser, K. L. Lunetta, Meta-analyses identify 13 loci associated with age at menopause and highlight DNA repair and immune pathways. Nat. Genet. 44, 260–268 (2012). 22267201 10.1038/ng.1051 PMC3288642

[48] D. Appiah, C. C. Nwabuo, I. A. Ebong, M. F. Wellons, S. J. Winters, Trends in age at natural menopause and reproductive life span among US women, 1959-2018. JAMA 325, 1328–1330 (2021). 33821908 10.1001/jama.2021.0278 PMC8025101

[49] D. J. Selkoe, Alzheimer’s disease is a synaptic failure. Science 298, 789–791 (2002). 12399581 10.1126/science.1074069

[50] S. Li, D. J. Selkoe, A mechanistic hypothesis for the impairment of synaptic plasticity by soluble Aβ oligomers from Alzheimer’s brain. J. Neurochem. 154, 583–597 (2020). 32180217 10.1111/jnc.15007 PMC7487043

[51] S. Tu, S. Okamoto, S. A. Lipton, H. Xu, Oligomeric Aβ-induced synaptic dysfunction in Alzheimer’s disease. Mol. Neurodegener. 9, 48 (2014). 25394486 10.1186/1750-1326-9-48 PMC4237769

[52] L. Zhou, J. McInnes, K. Wierda, M. Holt, A. G. Herrmann, R. J. Jackson, Y.-C. Wang, J. Swerts, J. Beyens, K. Miskiewicz, S. Vilain, I. Dewachter, D. Moechars, B. De Strooper, T. L. Spires-Jones, J. De Wit, P. Verstreken, Tau association with synaptic vesicles causes presynaptic dysfunction. Nat. Commun. 8, 15295 (2017). 28492240 10.1038/ncomms15295 PMC5437271

[53] A. M. Pooler, W. Noble, D. P. Hanger, A role for tau at the synapse in Alzheimer’s disease pathogenesis. Neuropharmacology 76, 1–8 (2014). 24076336 10.1016/j.neuropharm.2013.09.018

[54] T. L. Spires-Jones, B. T. Hyman, The intersection of amyloid beta and Tau at synapses in Alzheimer’s disease. Neuron 82, 756–771 (2014). 24853936 10.1016/j.neuron.2014.05.004 PMC4135182

[55] B. R. Hoover, M. N. Reed, J. Su, R. D. Penrod, L. A. Kotilinek, M. K. Grant, R. Pitstick, G. A. Carlson, L. M. Lanier, L.-L. Yuan, K. H. Ashe, D. Liao, Tau mislocalization to dendritic spines mediates synaptic dysfunction independently of neurodegeneration. Neuron 68, 1067–1081 (2010). 21172610 10.1016/j.neuron.2010.11.030 PMC3026458

[56] H. Depypere, A. Vergallo, P. Lemercier, S. Lista, A. Benedet, N. Ashton, E. Cavedo, H. Zetterberg, K. Blennow, E. Vanmechelen, H. Hampel, Neurodegeneration Precision Medicine Initiative (NPMI), Menopause hormone therapy significantly alters pathophysiological biomarkers of Alzheimer’s disease. Alzheimers Dement. 19, 1320–1330 (2023). 36218064 10.1002/alz.12759

[57] H. Shao, J. C. S. Breitner, R. A. Whitmer, J. Wang, K. Hayden, H. Wengreen, C. Corcoran, J. Tschanz, M. Norton, R. Munger, K. Welsh-Bohmer, P. P. Zandi, Cache County Investigators, Hormone therapy and Alzheimer disease dementia: New findings from the Cache County Study. Neurology 79, 1846–1852 (2012). 23100399 10.1212/WNL.0b013e318271f823 PMC3525314

[58] R. A. Whitmer, C. P. Quesenberry, J. Zhou, K. Yaffe, Timing of hormone therapy and dementia: The critical window theory revisited. Ann. Neurol. 69, 163–169 (2011). 21280086 10.1002/ana.22239 PMC3058824

[59] Y. Vinogradova, T. Dening, J. Hippisley-Cox, L. Taylor, M. Moore, C. Coupland, Use of menopausal hormone therapy and risk of dementia: Nested case-control studies using QResearch and CPRD databases. BMJ 374, n2182 (2021). 34588168 10.1136/bmj.n2182 PMC8479814

[60] D. A. Bennett, A. S. Buchman, P. A. Boyle, L. L. Barnes, R. S. Wilson, J. A. Schneider, Religious orders study and rush memory and aging project. J. Alzheimers Dis. 64, S161–S189 (2018). 29865057 10.3233/JAD-179939 PMC6380522

[61] S. Day, R. Mason, S. Lagosky, P. A. Rochon, Integrating and evaluating sex and gender in health research. Health Res. Policy Syst. 14, 75 (2016). 27724961 10.1186/s12961-016-0147-7 PMC5057373

[62] D. A. Bennett, J. A. Schneider, A. S. Buchman, L. L. Barnes, P. A. Boyle, R. S. Wilson, Overview and findings from the rush memory and aging project. Curr. Alzheimer Res. 9, 646–663 (2012). 22471867 10.2174/156720512801322663 PMC3439198

[63] V. E. Barakauskas, C. L. Beasley, A. M. Barr, A. R. Ypsilanti, H.-Y. Li, A. E. Thornton, H. Wong, G. Rosokilja, J. J. Mann, B. Mancevski, Z. Jakovski, N. Davceva, B. Ilievski, A. J. Dwork, P. Falkai, W. G. Honer, A Novel mechanism and treatment target for presynaptic abnormalities in specific striatal regions in schizophrenia. Neuropsychopharmacology 35, 1226–1238 (2010). 20072114 10.1038/npp.2009.228 PMC3055413

[64] D. A. Bennett, J. A. Schneider, R. S. Wilson, J. L. Bienias, S. E. Arnold, Neurofibrillary tangles mediate the association of amyloid load with clinical Alzheimer disease and level of cognitive function. Arch. Neurol. 61, 378–384 (2004). 15023815 10.1001/archneur.61.3.378

[65] K. B. Casaletto, E. Nichols, V. Aslanyan, S. M. Simone, J. S. Rabin, R. L. Joie, A. M. Brickman, K. Dams-O’Connor, P. Palta, R. G. Kumar, K. M. George, C. L. Satizabal, J. Schneider, J. Pa, Sex-specific effects of microglial activation on Alzheimer’s disease proteinopathy in older adults. Brain 145, 3536–3545 (2022). 35869598 10.1093/brain/awac257 PMC10233295

[66] B. Rosner, G. A. Colditz, Age at menopause: Imputing age at menopause for women with a hysterectomy with application to risk of postmenopausal breast cancer. Ann. Epidemiol. 21, 450–460 (2011). 21441037 10.1016/j.annepidem.2011.02.010 PMC3117219

[67] R. S. Wilson, P. A. Boyle, L. Yu, L. L. Barnes, J. Sytsma, A. S. Buchman, D. A. Bennett, J. A. Schneider, Temporal course and pathologic basis of unawareness of memory loss in dementia. Neurology 85, 984–991 (2015). 26311746 10.1212/WNL.0000000000001935 PMC4567465

[68] D. A. Bennett, J. A. Schneider, N. T. Aggarwal, Z. Arvanitakis, R. C. Shah, J. F. Kelly, J. H. Fox, E. J. Cochran, D. Arends, A. D. Treinkman, R. S. Wilson, Decision rules guiding the clinical diagnosis of Alzheimer’s disease in two community-based cohort studies compared to standard practice in a clinic-based cohort study. Neuroepidemiology 27, 169–176 (2006). 17035694 10.1159/000096129

[69] L. Yu, M. W. Lutz, J. M. Farfel, R. S. Wilson, D. K. Burns, A. M. Saunders, P. L. De Jager, L. L. Barnes, J. A. Schneider, D. A. Bennett, Neuropathologic features of TOMM40 ‘523 variant on late-life cognitive decline. Alzheimers Dement. 13, 1380–1388 (2017). 28624335 10.1016/j.jalz.2017.05.002 PMC5723540

[70] R. L. Wasserstein, N. A. Lazar, The ASA statement on p-values: Context, process, and purpose. Am. Stat. 70, 129–133 (2016).

[71] J. L. Shifren, M. L. S. Gass, NAMS Recommendations for Clinical Care of Midlife Women Working Group, The North American Menopause Society recommendations for clinical care of midlife women. Menopause 21, 1038–1062 (2014). 25225714 10.1097/GME.0000000000000319

[72] J. S. Rabin, E. Nichols, R. La Joie, K. B. Casaletto, P. Palta, K. Dams-O’Connor, R. G. Kumar, K. M. George, C. L. Satizabal, J. A. Schneider, J. Pa, A. M. Brickman, Cerebral amyloid angiopathy interacts with neuritic amyloid plaques to promote tau and cognitive decline. Brain 145, 2823–2833 (2022). 35759327 10.1093/brain/awac178 PMC9420012

[73] E. Nichols, A. M. Brickman, K. B. Casaletto, K. Dams-O’Connor, K. M. George, R. G. Kumar, P. Palta, J. S. Rabin, C. L. Satizabal, J. Schneider, J. Pa, R. La Joie, AD and non-AD mediators of the pathway between the APOE genotype and cognition. Alzheimers Dement. 19, 2508–2519 (2023). 36516004 10.1002/alz.12885 PMC10264550

