# Supplementary material for: The interplay between age at menopause and synaptic integrity on Alzheimer’s disease risk in women
Source: Sci Adv. 2025 Mar 5;11(10):eadt0757. doi: 10.1126/sciadv.adt0757 (PMC11881898; doi:10.1126/sciadv.adt0757)
Supplement: Supplementary file 1 — Figs. S1 to S3 Tables S1 to S4 [file sciadv.adt0757_sm.pdf]

Science Advances
AAAS

Supplementary Materials for

The interplay between age at menopause and synaptic integrity on Alzheimer's disease risk in women

Madeline Wood Alexander *et al.*

Corresponding author: Kaitlin B. Casaletto, kaitlin.casaletto@ucsf.edu

Sci. Adv. **11**, eadt0757 (2025)
DOI: 10.1126/sciadv.adt0757

This PDF file includes:

Figs. S1 to S3
Tables S1 to S4

Fig. S1. Distribution of age at menopause in all participants.

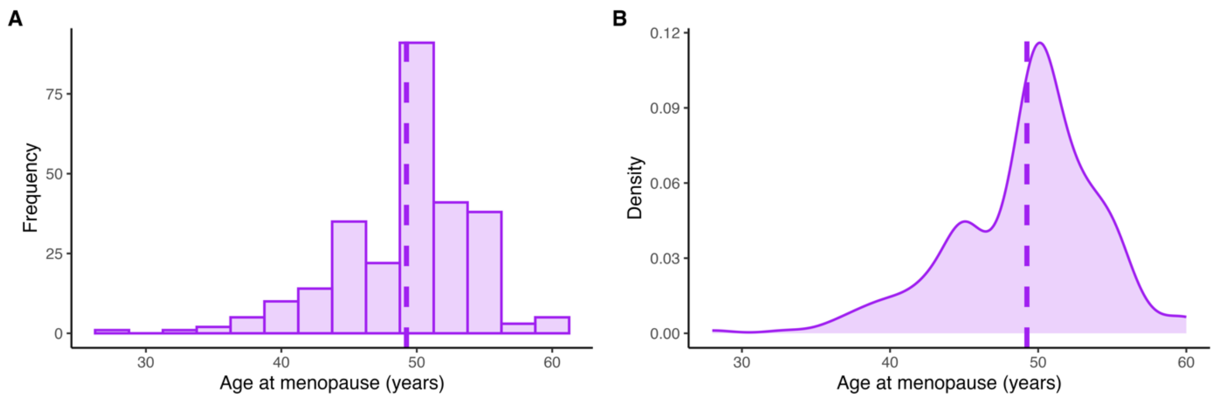

The plots depict the frequency (**A**) and density (**B**) of self-reported age at spontaneous menopause in the main analytic sample.

Fig. S2. Distributions of age at menopause stratified by history of hormone therapy.

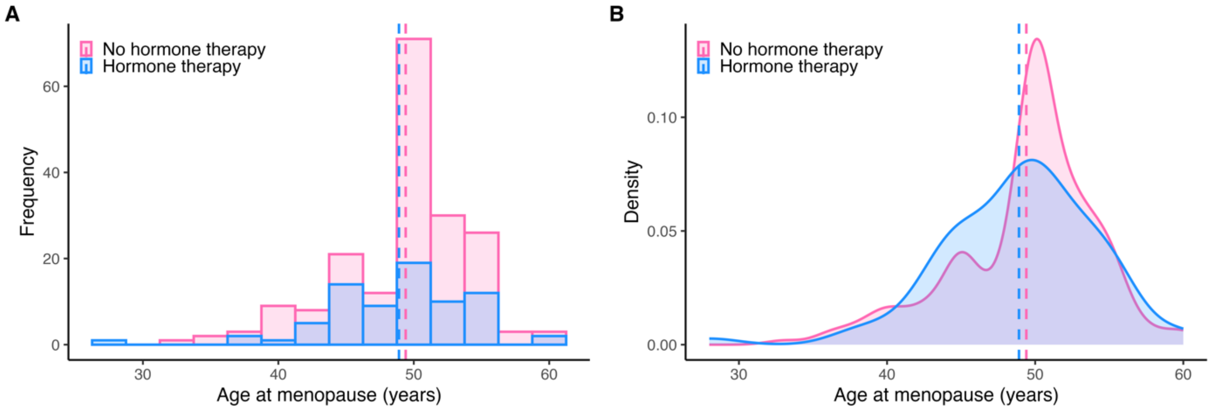

The plots depict the frequencies (**A**) and densities (**B**) of self-reported age at spontaneous menopause in the sample for exploratory hormone therapy analyses.

Fig. S3. Sample selection for main analyses.

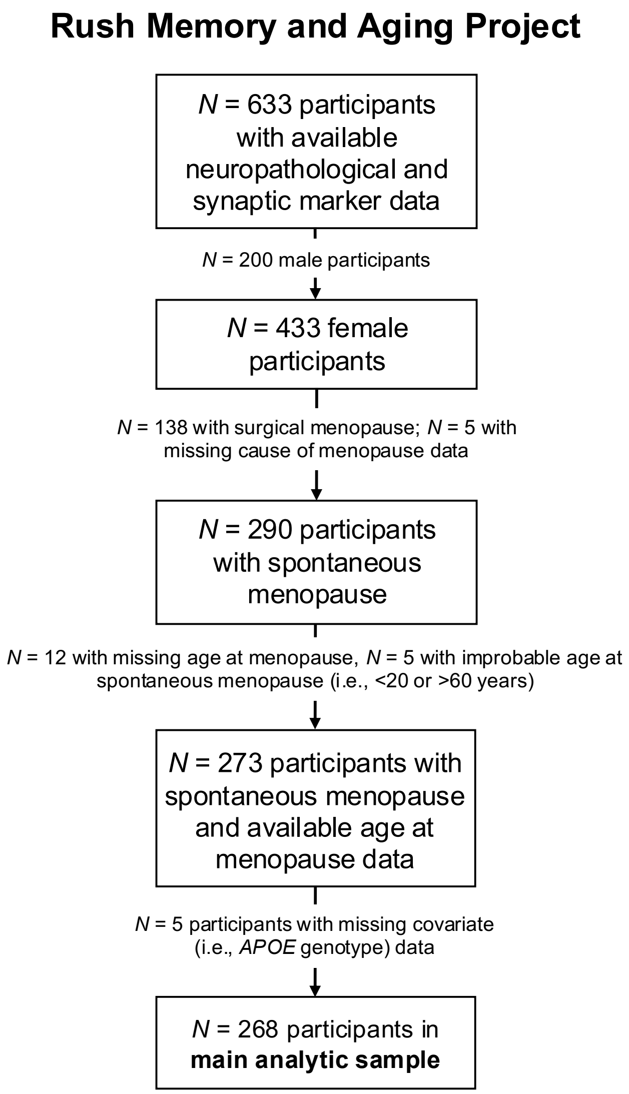

The flow chart depicts participant inclusions and exclusions made to form the main analytic sample.

Table S1.

Results of exploratory analyses testing direct associations of age at menopause with synaptic biomarkers, Alzheimer's disease (AD) neuropathology, and global cognitive decline in women with vs. without history of hormone therapy (HT). Synaptic biomarker and AD neuropathology models are adjusted for age at death, education, *APOE* ε4, β-amyloid (for tau model only), and mean SNARE density (for SNARE protein-protein interactions model only). Cognitive decline models are adjusted for the interaction between age at baseline and number of visits along with education, *APOE* ε4, mean SNARE density (for SNARE protein-protein interactions model only), and their interactions with time.

**Analyses**	**Women with no HT, *N* = 189**		**Women with history of HT, *N* = 75**	
	**β (95% CI)**	***p***	**β (95% CI)**	***p***
**Complexin-I ~ Age at menopause + covariates**				
Age at menopause	-0.005 (-0.029, 0.020)	.71	-0.016 (-0.055, 0.022)	.41
**Complexin-II ~ Age at menopause + covariates**				
Age at menopause	-0.0003 (-0.024, 0.023)	.98	-0.011 (-0.046, 0.024)	.54
**SNARE interactions ~ Age at menopause + covariates**				
Age at menopause	0.009 (-0.015, 0.034)	.46	-0.028 (-0.063, 0.007)	.11
**β-amyloid ~ Age at menopause + covariates**				
Age at menopause	-0.006 (-0.038, 0.026)	.69	-0.027 (-0.076, 0.021)	.26
**Tau ~ Age at menopause + covariates**				
Age at menopause	0.006 (-0.030, 0.042)	.75	0.012 (-0.031, 0.056)	.57
**Global cognition score ~ Age at menopause * time + covariates**				
Age at menopause	-0.002 (-0.007, 0.002)	.33	-0.001 (-0.008, 0.005)	.71

Table S2.

Results of exploratory analyses testing interactions of synaptic biomarkers and age at menopause on tau tangles in women with vs. without history of hormone therapy (HT). Models are adjusted for age at death, education, *APOE* ε4, β-amyloid, and mean SNARE density (for SNARE protein-protein interactions model only).

**Analyses**	**Women with no HT, *N* = 189**		**Women with history of HT, *N* = 75**	
	**β (95% CI)**	***p***	**β (95% CI)**	***p***
**Tau ~ complexin-I * age at menopause + covariates**				
Complexin-I * age at menopause	0.088 (0.039, 0.134)	.001	0.042 (-0.011, 0.095)	.12
**Tau ~ complexin-II * age at menopause + covariates**				
Complexin-II * age at menopause	0.061 (0.006, 0.117)	.03	-0.013 (-0.079, 0.054)	.70
**Tau ~ SNARE interactions * age at menopause + covariates**				
SNARE interaction * age at menopause	0.077 (0.032, 0.123)	.001	0.043 (-0.104, 0.018)	.17

Table S3.

Results of exploratory analyses testing interactions of synaptic markers and age at menopause on global cognitive decline in women with vs. without history of hormone therapy (HT). Models are adjusted for the interaction between age at baseline and number of visits along with education, *APOE* ε4, mean SNARE density (for SNARE protein-protein interactions model only), and their interactions with time.

**Analyses**	**Women with no HT, *N* = 179**		**Women with history of HT, *N* = 71**	
	**β (95% CI)**	***p***	**β (95% CI)**	***p***
**Global cognition score ~ complexin-I * age at menopause * time + covariates**				
Complexin-I * age at menopause * time	-0.011 (-0.018, -0.005)	.001	-0.004 (-0.012, 0.005)	.39
**Global cognition score ~ complexin-II * age at menopause * time + covariates**				
Complexin-II * age at menopause * time	-0.009 (-0.016, -0.002)	.02	0.001 (-0.009, 0.010)	.90
**Global cognition score ~ SNARE interactions * age at menopause * time + covariates**				
SNARE interactions * age at menopause * time	-0.008 (-0.014, -0.002)	.008	-0.001 (-0.010, 0.007)	.75

Table S4.

Results of sensitivity analyses additionally adjusting main models for common non-Alzheimer's disease neuropathologies. Cognitive models are adjusted for the interaction between age at baseline and number of visits along with education, *APOE* ε4, mean SNARE density (for SNARE protein-protein interactions model only), CAA, Lewy body disease, TDP-43, hippocampal sclerosis, vascular pathology, and their interactions with time. Neuropathological models are adjusted for age at death, education, *APOE* ε4, β-amyloid (for models where tau is the outcome), mean SNARE density (for SNARE protein-protein interactions model only), CAA, Lewy body disease, TDP-43, hippocampal sclerosis, and vascular pathology.

**Analyses**	**β (95% CI)**	***p***
**a) Synergistic associations of synaptic makers and age at menopause on cognitive decline.**		
Global cognition score ~ complexin-I * age at menopause * time + covariates, *N* = 240		
Complexin-I * age at menopause*time	-0.006 (-0.011 – -0.001)	.01
Global cognition score ~ complexin-II * age at menopause * time + covariates, *N* = 240		
Complexin-II * age at menopause*time	-0.005 (-0.011 – 0.001)	.07
Global cognition score ~ SNARE interactions * age at menopause * time + covariates, *N* = 240		
SNARE interactions * age at menopause * time	-0.005 (-0.010 – -0.001)	.02
**b) Synergistic associations of synaptic makers and age at menopause on tau tangles.**		
Tau ~ complexin-I * age at menopause + covariates, *N* = 252		
Complexin-I * age at menopause	0.055 (0.017 – 0.093)	.005
Tau ~ complexin-II * age at menopause + covariates, *N* = 252		
Complexin-II * age at menopause	0.017 (-0.029 – 0.062)	.47
Tau ~ SNARE interactions * age at menopause + covariates, *N* = 252		
SNARE interactions * age at menopause	0.033 (-0.003 – 0.070)	.08
**c) Synergistic associations of synaptic makers and age at menopause on β-amyloid.**		
β-amyloid ~ complexin-I * age at menopause + covariates, *N* = 252		
Complexin-I * age at menopause	0.023 (-0.011 – 0.057)	.18
β-amyloid ~ complexin-II * age at menopause + covariates, *N* = 252		
Complexin-II * age at menopause	0.015 (-0.026 – 0.055)	.48
β-amyloid ~ SNARE interactions * age at menopause + covariates, *N* = 252		
SNARE interactions * age at menopause	0.034 (0.001 – 0.066)	.04
